# Synergistic effects of biochar and *Bacillus subtilis* on soil fertility and tobacco leaf quality in acidified soil: microbial mechanisms and multi-model evaluation

**DOI:** 10.3389/fmicb.2026.1842613

**Published:** 2026-06-09

**Authors:** Weihua Hu, Zheng Wang, Qigang Yang, Tao Zhang, Bo Li, Jincheng Ao

**Affiliations:** 1 College of Resources and Environment, Yunnan Agricultural University, Kunming, Yunnan, China; 2 China Tobacco Guangxi Industrial Co., Ltd., Nanning, Guangxi, China; 3 College of Sanqi Medicine and Pharmacy, Wenshan University, Wenshan, Yunnan, China

**Keywords:** *Bacillus subtilis*, biochar, nitrogen cycling, soil acidification, soil bacterial community, tobacco leaf quality

## Abstract

Soil acidification severely constrains soil fertility and crop quality in tobacco-growing regions of southern China. Although biochar and beneficial microorganisms are widely used as soil amendments, their synergistic effects and underlying mechanisms remain insufficiently understood. In this field study, five treatments were established in acidified red soil: a control without biochar or microbial fertilizer (CK), rice husk biochar alone at 1500 kg·ha^−1^ (BF0), and biochar combined with *Bacillus subtilis* fertilizer at 15, 30, and 60 kg·ha^−1^, denoted as BF1, BF2, and BF3, respectively. The effects of these treatments on soil physicochemical properties, microbial communities, and tobacco leaf quality were systematically evaluated. High-throughput 16S rRNA sequencing revealed that biochar–microbe co-application significantly reshaped soil bacterial community structure and enhanced microbial diversity. Functional prediction indicated that moderate microbial input strengthened nitrogen cycling potential. These microbial responses were accompanied by improved soil physicochemical properties and enhanced activities of key enzymes involved in carbon and nitrogen transformation. A comprehensive evaluation based on principal component analysis showed that both BF1 and BF2 achieved the highest soil fertility levels, with no significant difference between them. Tobacco leaf quality was assessed using entropy-weighted grey relational analysis (GRA) and supported by TOPSIS, which consistently indicated that BF2 and BF3 performed best, also without significant difference. Considering both soil fertility and tobacco leaf quality, BF2 was identified as the optimal treatment, achieving a balanced improvement in soil conditions and leaf chemical composition. Partial least squares path modeling (PLS-PM) further revealed that biochar–microbe co-application enhanced soil fertility primarily through enzyme-mediated pathways, with enzyme activity exerting strong direct effects on soil fertility and indirect effects via microbial functional shifts. Improved soil fertility subsequently promoted tobacco growth, optimized leaf chemical composition, and ultimately enhanced overall leaf quality. Overall, these findings demonstrate that combined application of biochar and *B. subtilis* improves soil fertility and tobacco leaf quality through coupled physicochemical–microbial mechanisms, with moderate microbial input representing a balanced and efficient management strategy for acidified soils.

## 1 Introduction

Soil acidification is a widespread form of land degradation that threatens the sustainability of agricultural ecosystems worldwide. Its primary mechanism involves the long-term depletion of soil acid–base buffering capacity caused by continuous inputs of exogenous acidic substances, leading to elevated hydrogen ion concentrations, loss of base cations, and a progressive decline in soil pH (Zhang N. et al., 2025). These changes trigger a cascade of negative ecological consequences, including nutrient imbalance, increased availability of toxic ions such as aluminum and manganese, and disruption of soil microbial community structure and function, ultimately threatening crop productivity and agroecosystem stability (Wan et al., 2019; Ofoe et al., 2023).

In southern China, soil acidification has become particularly severe in intensively cultivated red soil regions (Shen et al., 2021). Continuous fertilizer application and acid deposition, together with abundant rainfall and its associated leaching and erosion processes, accelerate soil acidification and nutrient loss, ultimately leading to widespread soil quality degradation (Yang et al., 2018). Flue-cured tobacco (*Nicotiana tabacum* L.), an economically important crop in this region, is highly sensitive to changes in the rhizosphere environment (Huan et al., 2024). Progressive soil acidification not only limits tobacco growth but also disrupts nutrient balance and affects the accumulation and coordination of key chemical components in tobacco leaves, thereby reducing leaf quality and industrial value (Rao et al., 2021). Therefore, developing sustainable strategies to improve soil fertility while maintaining tobacco leaf quality has become a critical challenge for tobacco production in acidified soils.

Among various remediation approaches, both abiotic amendments and biological interventions have been proposed to alleviate soil acidification. Biochar, produced through the pyrolysis of agricultural and forestry residues, has been widely investigated for acidic soil remediation due to its porous structure, large specific surface area, and alkaline characteristics (Kamali et al., 2022; Zhang W. et al., 2025). Biochar application can neutralize soil acidity, enhance soil aggregation, increase cation exchange capacity, and improve nutrient retention, thereby enhancing soil physicochemical properties (Suo et al., 2021; Zhong et al., 2025). In parallel, functional microorganisms such as *Bacillus subtilis* regulate soil processes through more direct biological pathways. These microorganisms can secrete extracellular enzymes that accelerate organic matter decomposition and nutrient release (Su et al., 2020; Mahapatra et al., 2022), while suppressing soil-borne pathogens through nutritional and spatial competition (Amutuhaire et al., 2025), thereby contributing to nutrient cycling and biological disease control. However, biochar-based remediation is largely passive and often exhibits delayed effects, whereas the functional efficacy of microbial inoculants is frequently constrained by limited survival and colonization in complex soil environments (Zhang et al., 2021).

Increasing evidence suggests that the combined application of biochar and functional microbial inoculants can generate synergistic effects (Chen et al., 2023; Ren et al., 2025). Biochar can provide protective microsites and carbon sources for introduced microorganisms, while microbial metabolic activity can accelerate biochar aging and surface modification, jointly enhancing the efficiency and stability of soil ecological restoration (Guo et al., 2020; Iacomino et al., 2024). Despite the recognized synergistic effects, previous studies have largely focused on fixed application ratios, and the regulatory roles of critical factors, particularly microbial inoculation rates, remain insufficiently explored. In acidified tobacco-growing systems, it remains unclear how varying *B. subtilis* application rates combined with a constant biochar input influence soil bacterial communities, carbon and nitrogen cycling, and subsequent tobacco leaf quality. We hypothesized that biochar provides ecological niches that enhance the colonization efficiency of *B. subtilis*, thereby stimulating enzyme-mediated nitrogen cycling and ultimately improving soil fertility and tobacco leaf quality.

Therefore, this study focused on typical acidified tobacco-growing red soils in southern China. A field experiment was conducted under conventional fertilization with a fixed biochar application rate and a gradient of *B. subtilis* fertilizer inputs. The objectives were to: (1) evaluate the effects of combined biochar and microbial applications at different bacterial doses on soil physicochemical properties, enzyme activities, and bacterial community structure; (2) quantitatively assess changes in comprehensive soil fertility and integrated tobacco leaf quality and identify the optimal biochar–microbe combination; and (3) integrate microbial community data, functional prediction, and multivariate statistical analyses to elucidate the key microbial pathways through which biochar–microbe interactions enhance soil fertility and subsequently improve tobacco leaf quality.

## 2 Materials and methods

### 2.1 Study area and materials

The study area was located in Luoping County, Qujing City, eastern Yunnan Province, China (24.91° N, 104.44° E), with an elevation ranging between 1,288 and 1900 m above sea level. The region features a plateau monsoon climate, with a mean annual temperature of 15.1 °C, a mean annual precipitation of 1743.9 mm, and a mean annual relative humidity of 85% (Xiong et al., 2020). The tested soil was acidic red soil with a pH of 5.45, and its basic chemical properties are listed in Supplementary Table S1. The tested biochar was a commercially available rice husk biochar with an alkaline pH of 9.67, containing 31.28% organic matter, 4.83 g·kg^−1^ total N, 1.19 g·kg^−1^ total P, and 15.63 g·kg^−1^ total K; detailed properties are provided in Supplementary Table S2. The tested commercial *Bacillus subtilis* fertilizer (≥ 2 × 10^8^ CFU·g^−1^) was used as the microbial agent. The main tobacco cultivar used in the experiment was ‘Yunyan 87’, and the tobacco seedlings were provided by China Tobacco Guangxi Industrial Co., Ltd.

### 2.2 Experimental design

The field experiment included one control group (CK) and four biochar-based treatments (BF0, BF1, BF2, BF3). The control group received conventional fertilization without biochar or microbial fertilizer. All biochar-based treatments received a basal application of rice husk biochar at 1500 kg·ha^−1^, combined with *B. subtilis* fertilizer at 0 (BF0), 15 (BF1), 30 (BF2), and 60 (BF3) kg·ha^−1^. The *B. subtilis* fertilizer had a labeled concentration of 2 × 10^8^ CFU·g^−1^; corresponding to application rates of approximately 0 (BF0), 3 × 10^12^ (BF1), 6 × 10^12^ (BF2), and 1.2 × 10^13^ (BF3) CFU·ha^−1^, respectively.

The experiment was arranged in a randomized complete block design. The experimental area was divided into three blocks, each containing all five treatments (CK, BF0, BF1, BF2, BF3), which were randomly assigned to plots within each block. Thus, each treatment had three replicates in total (one per block), and each replicate plot covered an area of 66.7 m^2^. Guard rows (1 m wide) were set between adjacent blocks to minimize inter-block interference.

Both biochar and the bacterial fertilizer were applied as basal fertilizers at transplanting. They were placed into the planting holes and thoroughly mixed with the soil in each hole. A tobacco-specific basal fertilizer (N: P_2_O_5_: K_2_O = 12:12:25, organic matter ≥ 8.5%) was also applied at this time, following the local recommended practice. Topdressing was carried out 50–60 days after transplanting during intertillage and soil ridging, using a tobacco-specific topdressing fertilizer (N: P_2_O_5_: K_2_O = 15:0:33). The total fertilizer application followed a basal-to-topdressing mass ratio of 6:4, with a total nitrogen input of 105 kg·ha^−1^. Transplanting was conducted in early May 2024 at a planting density of 16,500 plants·ha^−1^.

### 2.3 Experimental procedure

#### 2.3.1 Soil sampling

Background soil sampling (pre-transplanting): Before tobacco transplanting, composite soil samples were prepared by mixing soils collected from five sampling points across the experimental site, with soil taken from the plow layer (5–20 cm depth) using a soil sampler, for the determination of baseline soil physicochemical properties.

Plow layer soil sampling (bud stage): At the tobacco bud stage (July 5, 2024), non-rhizosphere soil samples were collected from each treatment plot using a core sampler. Specifically, after removing surface litter, the core sampler was vertically inserted into the soil. The intact soil core was carefully extracted, wrapped in plastic film, and used for subsequent measurements of soil bulk density and porosity.

Rhizosphere soil sampling (bud stage): Concurrently, three uniformly growing tobacco plants were randomly selected from each plot and carefully excavated with their root systems intact. Rhizosphere soil was collected using the shaking method (Philippot et al., 2013). After gently shaking off the bulk soil, a sterile brush was used to collect the soil tightly adhering to the root surfaces into zip-lock bags and cryovials.

Sample processing and preservation: For each plot, rhizosphere soils collected from the three sampled plants were pooled to form one composite sample (approximately 200 g per composite sample) and thoroughly homogenized. Composite samples from different plots were kept separate for subsequent analyses. Subsamples for high-throughput sequencing were transferred into sterile cryovials, flash-frozen in liquid nitrogen, and stored at −80 °C until DNA extraction. Subsamples for the physicochemical properties and enzyme activities analyses were air-dried in a cool, well-ventilated indoor environment, and then ground and sieved for further analysis.

#### 2.3.2 Determination of soil physicochemical properties and enzyme activities

Soil physicochemical properties were determined according to standard methods described in Methods of Soil Analysis (Page, 1982). Soil bulk density (SBD) and porosity (SP) were determined by the core method using a stainless steel ring (100 cm^3^). Soil pH was measured potentiometrically at a soil-to-water ratio of 1:2.5. Organic matter (OM) content was determined using the potassium dichromate oxidation method. Total nitrogen (TN) was analyzed by the Kjeldahl method. Alkali-hydrolyzable nitrogen (AN) was measured using the alkali diffusion method. Available phosphorus (AP) was extracted and determined by the molybdenum-antimony colorimetric method. Available potassium (AK) was extracted and measured using flame photometry. Cation exchange capacity (CEC) was determined by the ammonium acetate exchange method at pH 7.0.

Soil enzyme activities were assayed as follows: urease (URE) activity was determined by the indophenol blue method, sucrase (SUC) activity by the 3,5-dinitrosalicylic acid method, nitrate reductase (NR) activity by a colorimetric method, and catalase activity (CAT) by the potassium permanganate titration method. Enzyme activities were expressed on a dry soil mass basis.

#### 2.3.3 High-throughput sequencing of soil bacteria

Soil microbiome high-throughput sequencing was performed by Majorbio Bio-Pharm Technology Co., Ltd. (Shanghai, China)^1^ following established protocols (Fazal et al., 2023). Briefly, 0.50 g of fresh soil (accurately weighed using an electronic balance with a precision of 0.001 g) was used for total genomic DNA extraction with the HiPure Soil DNA Mini Kit (Majorbio, China). The integrity of the extracted DNA was verified by 1% agarose gel electrophoresis.

The V3-V4 hypervariable region of the bacterial 16S rRNA gene was amplified from the diluted genomic DNA using the barcoded universal primers 338F (5´-ACTCCTACGGGAGGGCAGCA-3′) and 806R (5´-GGACTACHVGGGTWTCTAAT-3′). PCR amplification was conducted using TransStart Fast PFU DNA Polymerase, with each sample amplified in triplicate. The PCR products from the three replicates were pooled and purified via 2% agarose gel electrophoresis. Sequencing libraries were constructed using the TruSeq DNA PCR-Free Library Preparation Kit (Illumina). After quality control and quantification using Qubit, the qualified libraries were sequenced on an Illumina NovaSeq 6,000 platform (paired-end, 2 × 250 bp).

#### 2.3.4 Determination of tobacco agronomic traits

At the topping stage of flue-cured tobacco (July 15), the agronomic traits were measured according to the industry standard Method for Investigation and Measurement of Tobacco Agronomic Traits (YC/T 142–2010) issued by the State Tobacco Monopoly Administration. For each treatment, five plants were randomly selected, and their traits were measured using a soft tape measure (precision: 0.01 cm). The mean value was then calculated.

#### 2.3.5 Determination of leaf chemical composition

After topping and sucker control, tobacco leaves from each plot were harvested and cured separately. Following curing, approximately 2.0 kg of cured leaves of the middle stalk position (grade C3F) were randomly sampled per plot.

Total volatile bases (TVB) were determined by steam distillation. The other key chemical components of tobacco leaves, including nicotine (NIC), total sugar (TS), reducing sugar (RS), tobacco total nitrogen (TTN), potassium (K), and chlorine (Cl), were determined using near-infrared spectroscopy (NIRS). The NIRS prediction models used for chemical component analysis were developed by the College of Energy and Environmental Science, Yunnan Normal University, and have been applied in previous studies (Li et al., 2022; Wang et al., 2026). All NIRS measurements in this study were conducted by the same research group. Briefly, the prepared powder samples were placed in sample cups, and their spectra were collected using an NIRS spectrometer equipped with an integrating sphere in the wavelength range of 800–2,500 nm. Each recorded spectrum was the average of 32 scans to minimize noise.

The acquired spectral data were then processed using a dedicated, tobacco-validated multivariate calibration model to predict the content of each chemical component. Based on the predicted values, the following quality indices were calculated: sugar-to-nicotine ratio (TS/NIC), nitrogen-to-nicotine ratio (TTN/NIC), potassium-to-chloride ratio (K/Cl), and reducing-to-total sugar ratio (RS/TS).

### 2.4 Bioinformatics and statistical analysis

All measured data were organized and recorded using Excel 2024. The mean and standard deviation were calculated for each treatment. Prior to statistical analysis, data were tested for normality using the Shapiro–Wilk test and for homogeneity of variances using Levene’s test. One-way analysis of variance (ANOVA) was performed using SPSS Statistics 27.0 (IBM Corp., Armonk, NY, USA). When the assumptions were satisfied, Duncan’s multiple range test was employed to assess significant differences among treatments at significance levels of *p* < 0.05 (significant) and *p* < 0.01 (highly significant). In addition to *p* values, 95% confidence intervals for effect sizes were calculated to better characterize treatment effects.

For high-throughput sequencing data, demultiplexed paired-end reads were processed in QIIME 2 (version 2025.4). Primer and adaptor sequences were removed, and reads were quality-filtered and denoised using the DADA2 plugin to infer amplicon sequence variants (ASVs), including dereplication and chimera removal. To minimize biases caused by uneven sequencing depth, the ASV feature table was normalized by rarefaction to the minimum sequencing depth across samples. Taxonomic assignment of representative ASV sequences was performed against the SILVA database (Release 138).

Microbial diversity was comprehensively evaluated using multiple alpha diversity indices, including Shannon, Sobs, Chao1, and phylogenetic diversity (PD). Beta diversity was assessed based on Bray–Curtis dissimilarity, and differences in community composition among treatments were visualized using non-metric multidimensional scaling (NMDS). Statistical significance of community dissimilarities was tested using ANOSIM.

Differential abundance analysis of bacterial taxa was conducted using the microeco package in R (version 4.4.2), incorporating linear discriminant analysis effect size (LEfSe) with an LDA threshold of >3. To control for potential false positives, *p* values were adjusted using the Benjamini–Hochberg false discovery rate (FDR) correction, and taxa with adjusted *p* < 0.05 were considered significantly different. Functional potentials of microbial communities were further predicted based on FAPROTAX, COG classification, and KEGG metabolic pathways.

### 2.5 Comprehensive soil fertility evaluation method

To comprehensively evaluate soil fertility, 14 indicators were selected based on their relevance to soil fertility and responsiveness to treatments, including soil chemical properties (pH, TN, AN, AP, AK, CEC), physical properties (SBD, SP), enzyme activities (NR, SUC), and the relative abundances of dominant bacterial phyla (Proteobacteria, Actinobacteriota, Acidobacteriota, Chloroflexi). These variables represent key physicochemical and biological processes and showed significant variation among treatments or relatively high abundance in the microbial community.

All variables were standardized (Z-score) before analysis. Principal component analysis (PCA) was then applied to reduce dimensionality and integrate these indicators into a comprehensive soil fertility index. Principal components with eigenvalues > 1 were retained, and their scores were combined into a single fertility score using the proportion of variance explained by each retained component as weights.

Mantel and Pearson correlation analyses were first used to explore relationships among soil properties and dominant bacterial phyla. All multivariate analyses were performed in R 4.4.2, mainly using the packages linkET, stats, and ggplot2. Multiple multivariate analyses were used to integrate soil physicochemical, enzymatic, and bacterial community indicators for a more robust evaluation of soil fertility. The principal component loadings are provided in Supplementary Figure S1. Based on the loadings of each indicator on the retained principal components, the comprehensive soil fertility index was calculated for each treatment group according to Equation 1.


F=0.400PC1+0.232PC2+0.145PC3+0.107PC4 (1)

### 2.6 Comprehensive evaluation method of tobacco leaf quality

Tobacco leaf quality was evaluated using 14 indicators in this study, including three significant agronomic traits (Plant height, Leaf number, Leaf area), as well as conventional chemical composition and balance-related indices, comprising seven conventional chemical components (TS, RS, TTN, NIC, TVB, K, Cl), and four conventional derived indices characterizing compositional harmony (K/Cl, RS/TS, TS/NIC, TTN/NIC). Data preprocessing was conducted in R using the dplyr package.

**Figure 1:**
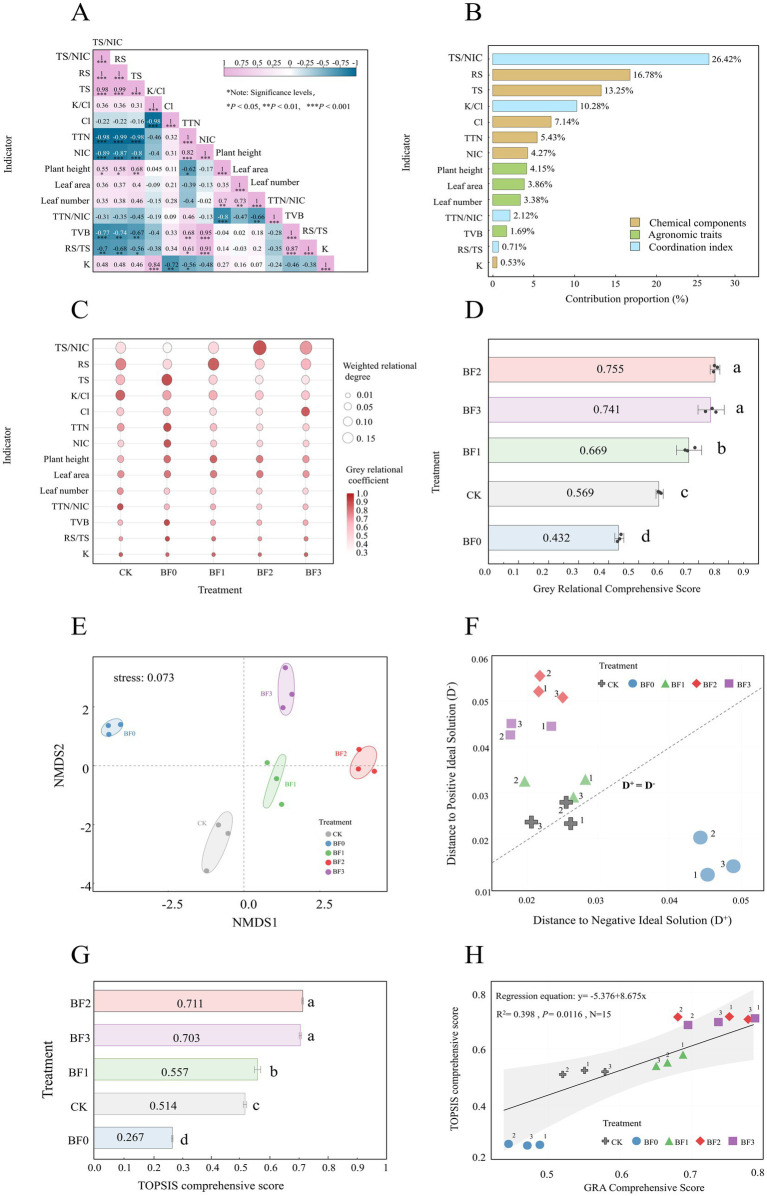
Comprehensive analysis of tobacco leaf quality. Indicator correlation and weights: **(A)** Heatmap of correlations among tobacco leaf quality indicators, **(B)** Bar chart of indicator weights ranked by the entropy weight method. Grey relational analysis **(C)** Bubble chart of GRA, **(D)** Ranking chart of GRA scores. TOPSIS analysis: **(E)** NMDS distribution plot of tobacco leaf indicators, **(F)** TOPSIS decision space plot, **(G)** Ranking chart of TOPSIS scores. GRA-TOPSIS linear correlation validation: **(H)** Linear regression fitting between GRA and TOPSIS analysis.

The entropy weights of all indicators used in grey relational analysis (GRA) and the Technique for Order Preference by Similarity to Ideal Solution (TOPSIS) (see Figure 1B), together with their optimal ranges and reference values, are listed in Supplementary Table S3. For optimal-range indicators, the reference value was defined as the midpoint of the accepted optimal range for high-quality flue-cured tobacco, with the range itself established based on quality standards reported in previous studies and industry guidelines in China (Chen et al., 2017; Chang et al., 2023). Indicators were classified into three types according to their contribution to quality:

(i) Larger-the-better: plant height, leaf number, leaf area, K, K/Cl, RS/TS;
(ii) Smaller-the-better: Cl;
(iii) Optimal-range: TS, RS, TTN, NIC, TVB, TS/NIC, TTN/NIC.

#### 2.6.1 Entropy weighting

Indicator weights were determined objectively using the entropy weight method, the weight *w_j_* for the *j*-th indicator was calculated as Equation 2.


$$E_{j}=-\frac{1}{\ln m}\sum_{i=1}^{n}p_{\mathrm{ij}}\ln(p_{\mathrm{ij}}),w_{i}=\frac{1-E_{j}}{\sum_{j=1}^{n}{\left(1-E_{j}\right)}^{'}}$$ (2)

where *p_ij_* is the proportion of the *i*-th sample under the *j*-th indicator, *m* is the number of samples, and *n* is the number of indicators.

#### 2.6.2 Grey relational analysis (GRA)

An entropy-weighted GRA model was used as the primary evaluation method (Hu et al., 2022). The ideal reference sequence *X_0_* was defined based on indicator type: maximum values for larger-the-better indicators, minimum values for the smaller-the-better indicator, and optimal midpoints for optimal-range indicators. Mean values were normalized for all data.

The grey relational coefficient was calculated as Equation 3:


$$Y_{\mathrm{ij}}\equiv \frac{\min_{i}\min_{j}\Delta_{\mathrm{ij}}+\rho \cdot \max_{i}\max_{j}\Delta_{\mathrm{ij}}}{\Delta_{\mathrm{ij}}+\rho \cdot \max_{i}\max_{j}\Delta_{\mathrm{ij}}}$$ (3)

where *Δ_ij_* = ∣*y_ij_* − *y_0j_*∣ is the absolute difference between the normalized value *y_ij_* of the *i*-th sample and the normalized ideal value *y_0j_* for the *j*-th indicator, and *ρ* = 0.5 is the distinguishing coefficient.

The comprehensive relational degree *R_i_* (comprehensive score) for the *i*-th sample was then calculated as Equation 4.


$$R_{i}\equiv \sum_{j=1}^{n}W_{j}\cdot \gamma_{\mathrm{ij}}$$ (4)

#### 2.6.3 Entropy-weighted TOPSIS

As a supplementary validation, an entropy-weighted TOPSIS model was applied using the same indicator set and weights. All indicators were first homogenized into benefit-type variables: reciprocal transformation was applied to the smaller-the-better indicator (Cl), and optimal-range indicators were transformed using Equation 5. Data were then standardized (Z-score) and weighted according to Equation 6.


$$X^{\prime }=\frac{1}{1+|X-X_{0}|}$$ (5)

$$v_{\mathrm{ij}}=w_{j}z_{\mathrm{ij}}$$ (6)

The positive ideal solution (*V^+^*) and negative ideal solution (*V^−^*) were defined by Equations 7, 8.


$$v_{j}^{+}\equiv \max_{1\leq i\leq m}\{v_{\mathrm{ij}}\},V^{+}=(v_{1}^{+},v_{2}^{+},\ldots ,v_{n}^{+}),$$ (7)

$$v_{j}^{-}\equiv \min_{1\leq i\leq m}\{v_{\mathrm{ij}}\},V^{-}=(v_{1}^{-},v_{2}^{-},\ldots ,v_{n}^{-})$$ (8)

The Euclidean distances from each sample to the positive ideal solution (*d^+^_i_*) and to the negative ideal solution (*d^−^_i_*) were calculated as Equation 9.


$$d_{i}^{+}\equiv \sqrt{\sum_{j=1}^{n}{\left(v_{\mathrm{ij}}-v_{j}^{+}\right)}^{2},d_{i}^{-}}\equiv \sqrt{\sum_{j=1}^{n}{\left(v_{\mathrm{ij}}-v_{j}^{-}\right)}^{2}}$$ (9)

Finally, the relative closeness coefficient *C_i_* for the *i*-th sample was calculated as Equation 10.


$$C_{i}\equiv \frac{d_{i}^{-}}{d_{i}^{+}+d_{i}^{-}}$$ (10)

Higher *R_i_* and *C_i_* values indicate better tobacco leaf quality.

## 3 Results and analysis

### 3.1 Soil physicochemical properties and enzyme activities

**Figure 2:**
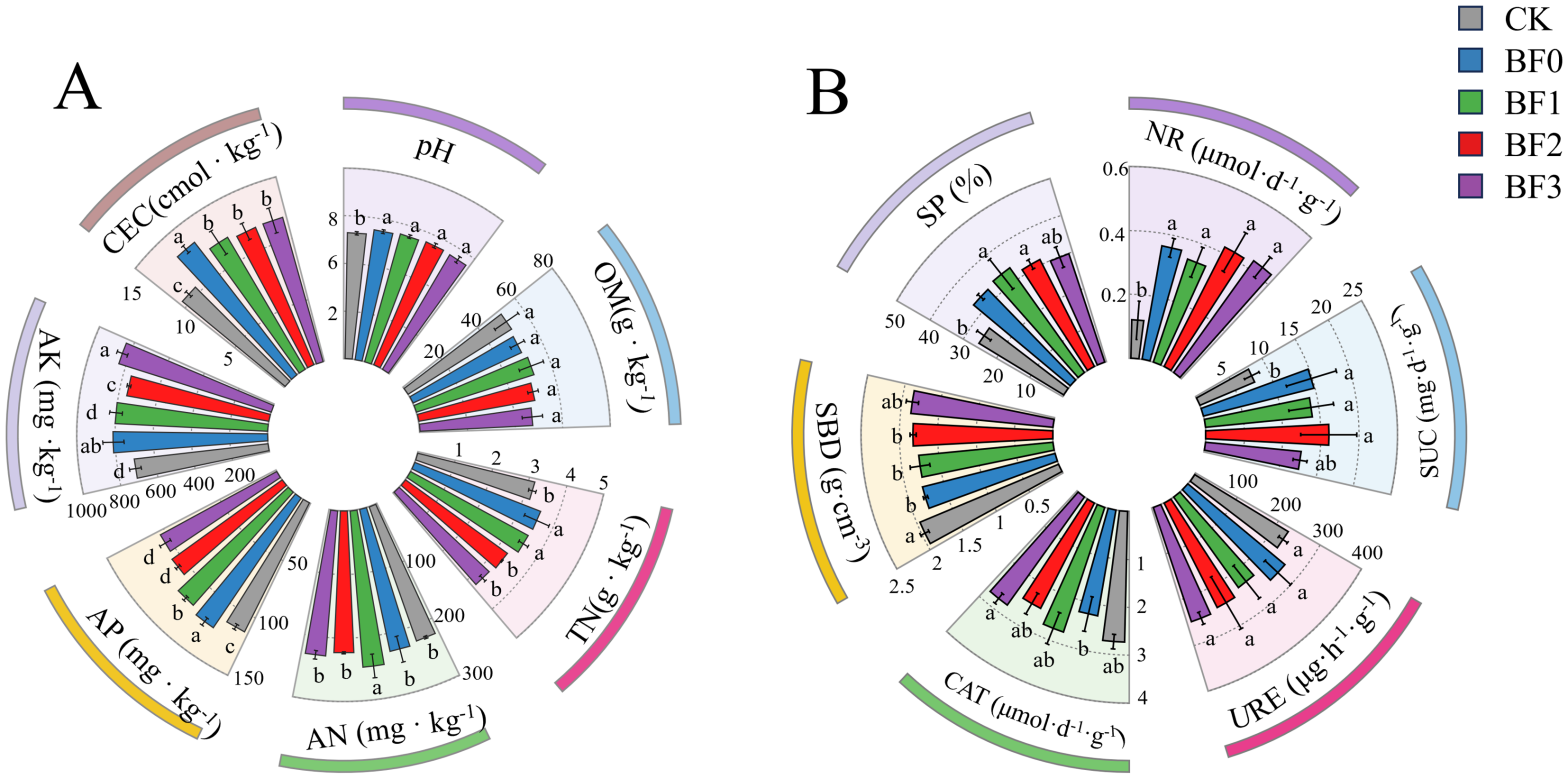
Effects of different biochar and *Bacillus subtilis* co-application modes on the physicochemical properties and enzyme activities of red soil: **(A)** Soil chemical properties, including cation exchange capacity (CEC), pH, organic matter (OM), total nitrogen (TN), alkali-hydrolyzable nitrogen (AN), available phosphorus (AP), and available potassium (AK). **(B)** Soil physical properties and enzyme activities, including bulk density (SBD), porosity (SP), catalase (CAT), urease (URE), sucrase (SUC), and nitrate reductase (NR). Different lowercase letters indicate significant differences among treatments at *p* < 0.05.

The co-application of biochar and *B. subtilis* substantially altered soil physicochemical properties and enzyme activities, as summarized in Figure 2. Regarding soil chemical properties (Figure 2A), compared with the control (CK), the application of biochar alone (BF0) and the combined application of biochar with microbial fertilizer (BF1-BF3) significantly increased soil pH (*p* < 0.05). The treatments also showed positive effects on TN, AK, and CEC. Specifically, BF0 and BF1 significantly increased TN (*p* < 0.05), while BF1 also significantly increased AN. Differences among treatments were also observed for AP, AK, and CEC. BF0 and BF1 significantly increased AP, whereas BF2 and BF3 significantly decreased AP (*p* < 0.05). All treatments significantly increased AK and CEC (*p* < 0.05). The increase in AK was 6.93% under BF2, while the other treatments exceeded 13.24%. The increase in CEC ranged from 12.19 to 25.52% across treatments. Overall, BF1 showed the most favorable effects on soil chemical properties.

Regarding soil physical properties and enzyme activities (Figure 2B), changes in soil physical properties and enzyme activities are presented in Figure 2B. Compared with CK, all treatments (BF0-BF3) reduced SBD and increased SP. The reduction in SBD ranged from 5.10 to 10.20%, while SP increased by 14.92 to 29.57%. Among them, BF0, BF1, and BF2 showed significant differences in both SBD reduction and SP increase (*p* < 0.05). In terms of enzyme activities, all treatments increased the URE, NR, and SUC to varying degrees compared with CK. URE activity increased by 1.63–15.02% under BF0-BF3, but the differences among treatments were not significant (*p* > 0.05). In contrast, NR activity was significantly enhanced under all treatments (*p* < 0.01), with increases exceeding 183.33%. SUC activity was significantly increased by BF0, BF1, and BF2 (*p* < 0.05), with the highest value observed under BF2.

### 3.2 Soil bacterial community structure

**Figure 3:**
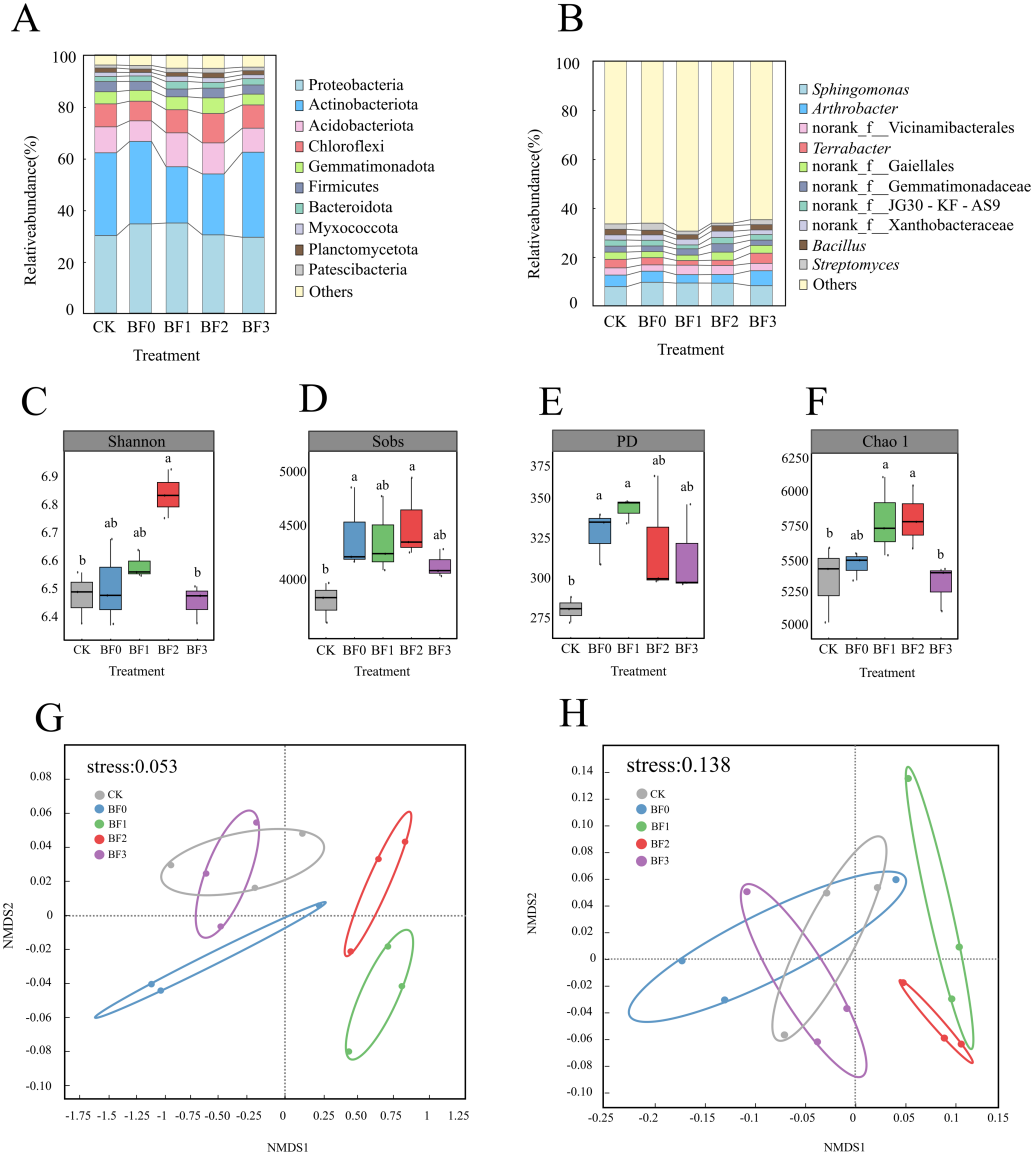
Soil bacterial community structure. Bacterial composition: **(A)** Relative abundance of dominant phyla, **(B)** Relative abundance of dominant genera ordination plot. Bacterial alpha diversity: **(C)** Shannon index, **(D)** Sobs index, **(E)** Phylogenetic diversity (PD) whole tree index, **(F)** Chao1 index. Bacterial beta diversity: **(G)** Non-metric multidimensional scaling (NMDS) ordination plot at the phylum level, **(H)** NMDS ordination plot at the genus level.

Sequencing generated sufficient depth to characterize bacterial diversity across treatments. Following taxonomic annotation, 10 major bacterial phyla were identified for analysis (Figures 3A,B). The dominant phyla were Proteobacteria (30.28–35.54%), Actinobacteriota (22.29–33.62%), Acidobacteriota (8.13–13.41%), and Chloroflexi (7.81–11.66%). Their cumulative relative abundance exceeded 78% in all treatment groups. Among these, the relative abundance of Actinobacteriota varied considerably. Relative to 32.66% in the CK treatment, it increased by 0.96% under BF3, while it decreased by 0.07, 10.37, and 8.56% in the BF0, BF1, and BF2 treatments, respectively.

A large proportion of taxa remained unclassified at the genus level. *Sphingomonas* (7.82–9.71%), *Arthrobacter* (3.55–6.17%) and *Terrabacter* (1.94–4.22%) had a cumulative relative abundance ranging from 14.84 to 18.51% across all treatments. Among these, *Sphingomonas* and Terrabacter underwent substantial changes in relative abundance. Relative to the CK treatment (*Sphingomonas*: 7.82%; *Terrabacter*: 3.58%), the BF0 to BF3 treatments increased the relative abundance of *Sphingomonas* by 0.38 to 24.17%, while the BF0 to BF2 treatments reduced the relative abundance of *Terrabacter* by 18.72 to 45.81%.

Regarding bacterial alpha diversity (Figures 3C–F), Good’s coverage of all samples exceeded 95%, indicating reliable sequencing data. Compared with CK, co-application of biochar and bacterial fertilizer modified bacterial alpha diversity: BF2 significantly increased the Shannon and Chao1 indices (*p* < 0.05), while BF3 significantly decreased both indices (*p* < 0.05). The PD index was significantly increased in BF0 and BF1 (*p* < 0.05), with BF2 and BF3 showing intermediate values. The Chao1 index was significantly higher in BF1 and BF2 (*p* < 0.05), while CK and BF3 remained at lower levels.

Non-metric multidimensional scaling (NMDS) analysis showed that biochar–microbe co-application altered soil bacterial community structure (Figures 3G,H). At the phylum level (stress = 0.053), BF0–BF2 were clearly separated from CK, with minimal overlap for BF3. At the genus level (stress = 0.138), the separation was less distinct, yet all treatments still diverged from CK, confirming significant community shifts at both taxonomic levels.

### 3.3 Comprehensive evaluation of soil fertility

**Figure 4:**
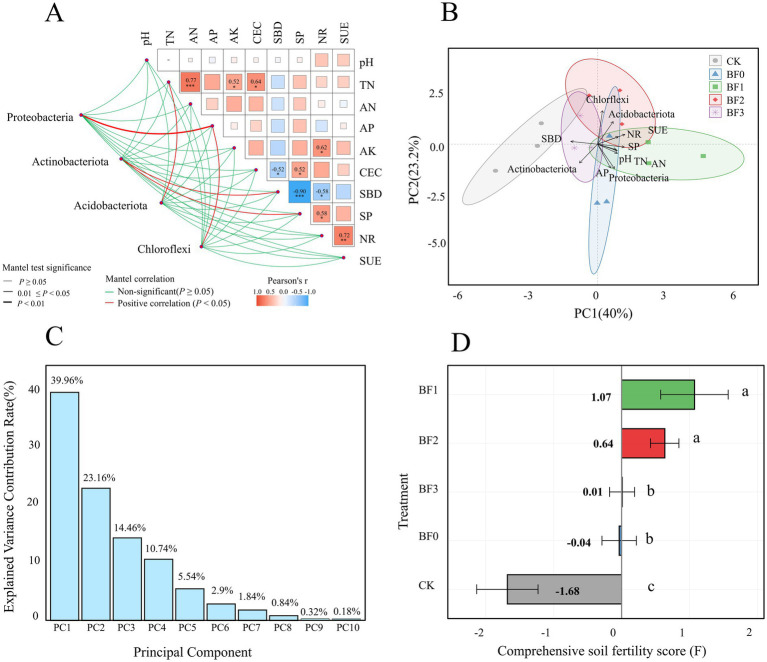
Comprehensive evaluation of soil fertility. Relationships between environmental factors and microorganisms: **(A)** Mantel test correlogram between dominant bacterial phyla and environmental factors, **(B)** Principal component analysis (PCA) biplot of dominant phyla and environmental factors. Integrated soil environmental assessment: **(C)** Variance explained by principal components, **(D)** Comprehensive soil fertility score.

Mantel test and Pearson correlation analysis (Figure 4A) revealed certain correlations between the dominant bacterial phyla and soil environmental factors, with most correlations being low to moderate. Proteobacteria showed a highly significant positive correlation (*p* < 0.01) with AP. Actinobacteriota was significantly positively correlated (*p* < 0.05) with TN. Acidobacteriota exhibited significant positive correlations (*p* < 0.05) with SBD and SP. Chloroflexi was significantly positively correlated (*p* < 0.05) with AP.

Given the relatively low intercorrelation among the selected soil indicators, PCA was deemed suitable for soil fertility evaluation. The PCA biplot (Figure 4B), with PC1 (40.0%) and PC2 (23.2%) collectively explaining 63.2% of the variance, revealed distinct spatial separation among the treatment groups based on their soil microbial and environmental indicators. The BF1 and BF2 groups were oriented toward the directions with higher explanatory power on the PC1 and PC2 axes, whereas the CK group was positioned in the area of lower explanatory power on both axes.

To comprehensively evaluate the soil fertility of each treatment, a PCA-based evaluation was performed (Figures 4C,D). Ten principal components were initially obtained, from which four with eigenvalues greater than 1 were extracted. These four components had a cumulative variance contribution rate of 88.32%, adequately representing the soil fertility information. A comprehensive scoring model (F) was subsequently established. The results demonstrated that all treatments significantly (*p* < 0.05) enhanced the comprehensive soil fertility score compared to CK. The overall ranking of soil fertility across treatments was BF1 > BF2 > BF3 > BF0 > CK. However, no significant difference was observed between BF1 and BF2, indicating that both treatments achieved similarly high soil fertility levels.

### 3.4 Differential analysis, functional prediction, and metabolic profiling of soil bacteria

**Figure 5:**
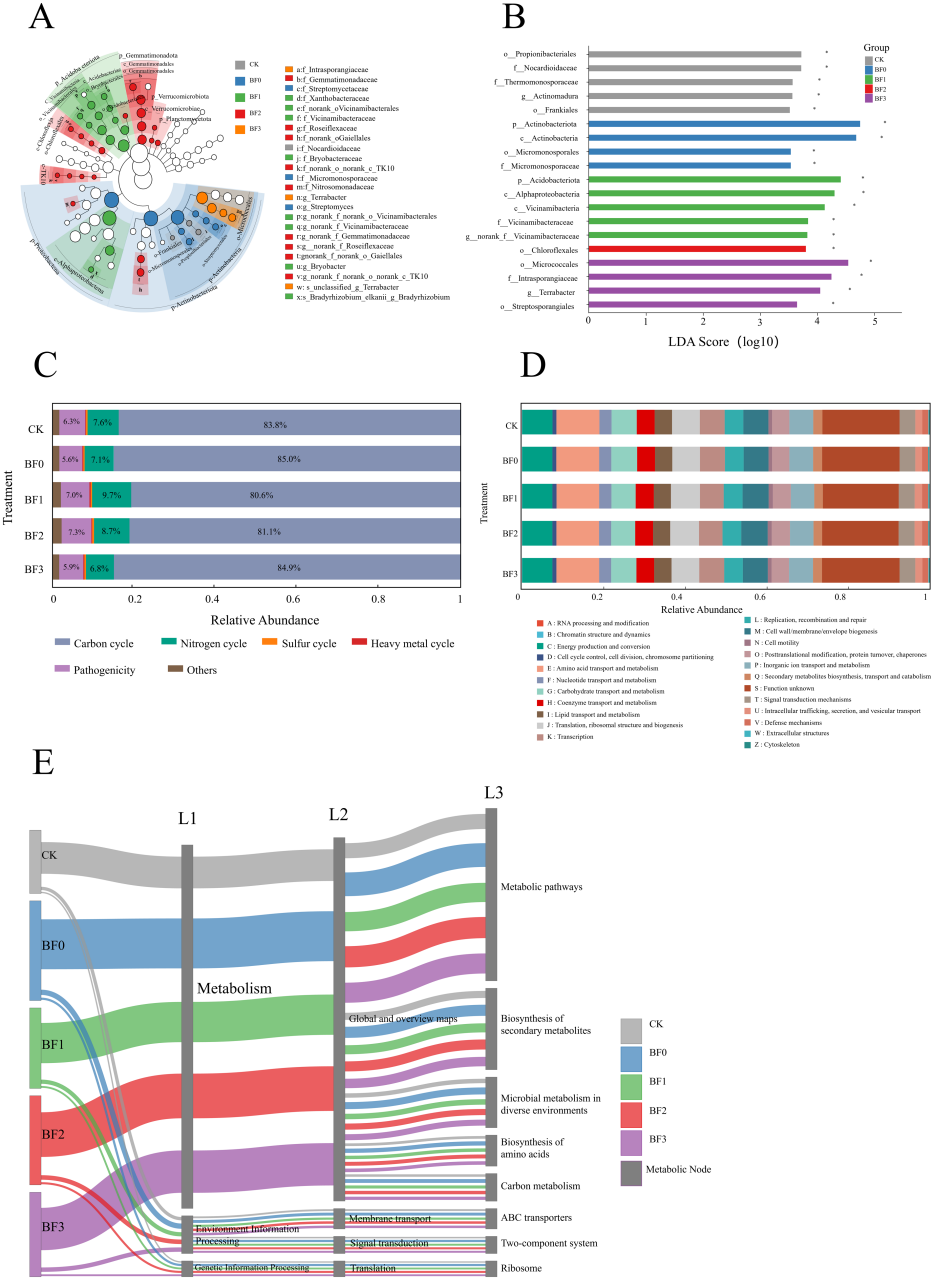
Bacterial function and metabolism. LEfSe analysis: **(A)** Cladogram, **(B)** Histogram of linear discriminant analysis (LDA) scores. Functional and metabolic pathway predictions: **(C)** FAPROTAX annotation, **(D)** COG functional annotation, **(E)** Sankey diagram of KEGG level-3 metabolic pathways.

Linear discriminant analysis effect size (LEfSe) revealed significant differences in bacterial taxa among treatments. The cladogram (Figure 5A) illustrates the phylogenetic relationships and group distribution of bacteria from phylum to family level, showing that differentially abundant taxa were primarily concentrated within six phyla: Actinobacteriota, Proteobacteria, Acidobacteriota, Gemmatimonadota, Verrucomicrobiota, and Planctomycetota, which were predominantly associated with the BF0, BF1, and BF2 treatment groups. The histogram of linear discriminant analysis (LDA) scores (Figure 5B) indicated that taxa with LDA scores > 3 all originated from the Actinobacteriota and Acidobacteriota phyla. This suggests that the treatments significantly altered the bacterial community compared to CK, primarily by modifying the abundances of these six bacterial phyla.

Functional annotation of soil bacteria using FAPROTAX identified 66 functional groups, which were categorized into six major functional guilds (Figure 5C). Carbon (C) and nitrogen (N) cycling processes exhibited the highest total relative abundance, accounting for over 90% in all treatments, representing the core ecological functions. Compared to CK, both BF1 and BF2 showed a decrease in C-cycling functions alongside an increase in N-cycling functions, with BF1 achieving the highest N-cycling abundance, significantly (*p* < 0.05) greater than other treatments. The BF1 treatment had microbial functions related to N-cycling and detoxification that were predicted to be enriched, while the BF2 treatment had functions associated with sulfur cycling and plant-microbe interactions (encompassing both pathogenic and symbiotic potentials) that showed higher relative abundance.

Annotation based on Clusters of Orthologous Groups of proteins (COG) identified 23 functional subcategories (Figure 5D). Among the four major COG categories—Information Storage and Processing; Cellular Processes and Signaling; Metabolism; and Poorly Characterized—Metabolism (encompassing subcategories C, G, E, F, H, I, P, Q) was the predominant functional category, comprising over 40% (range: 43.18–43.84%) in all treatments. Subcategories such as E (Amino acid transport and metabolism), C (Energy production and conversion), and G (Carbohydrate transport and metabolism) were also abundant, indicating high potential within the bacterial community for energy metabolism, carbon/nitrogen cycling, and secondary metabolism. The relative abundance of the Cellular Processes and Signaling category varied more notably among treatments (range: 19.58–20.56%). Within this category, subcategories M (Cell wall/membrane/envelope biogenesis) and O (Posttranslational modification, protein turnover, chaperones) were the top two in abundance, with BF1 and BF2 showing the highest values compared to CK, while BF3 showed a decrease. The Information Storage and Processing category accounted for 17.49–17.65% across groups, with BF2 being the highest.

The Sankey diagram (Figure 5E), based on KEGG pathway prediction with a minimum relative abundance threshold of 0.006%, illustrates key metabolic flows. It integrates 412 level-3 (L3) pathways classified into 46 level-2 (L2) pathways and 6 level-1 (L1) categories. The diagram highlights the most abundant core metabolic flows, including 3 L1 categories (Metabolism, Environmental Information Processing, Genetic Information Processing), 4 L2 categories (Membrane transport, Signal transduction, Translation, Global and overview maps), and the 7 most abundant L3 pathways across treatments. These represented core functional modules, collectively constituting 44% of the total predicted functions and reflecting primary treatment response trends. At the L2 level, “Global and overview maps” served as the widest downstream node, receiving input from all treatment groups via broad connecting lines, indicating consistently high activity of fundamental metabolic functions across treatments. “Environmental Information Processing” primarily received substantial input from the BF0 and BF2 treatments, suggesting that these treatments might influence microbial functionality by modulating pathways involved in environmental signal sensing and transduction. The varying widths of the connecting lines indicate distinct treatment effects on a specific metabolic pathway.

### 3.5 Agronomic traits and chemical composition of tobacco leaves

**Figure 6:**
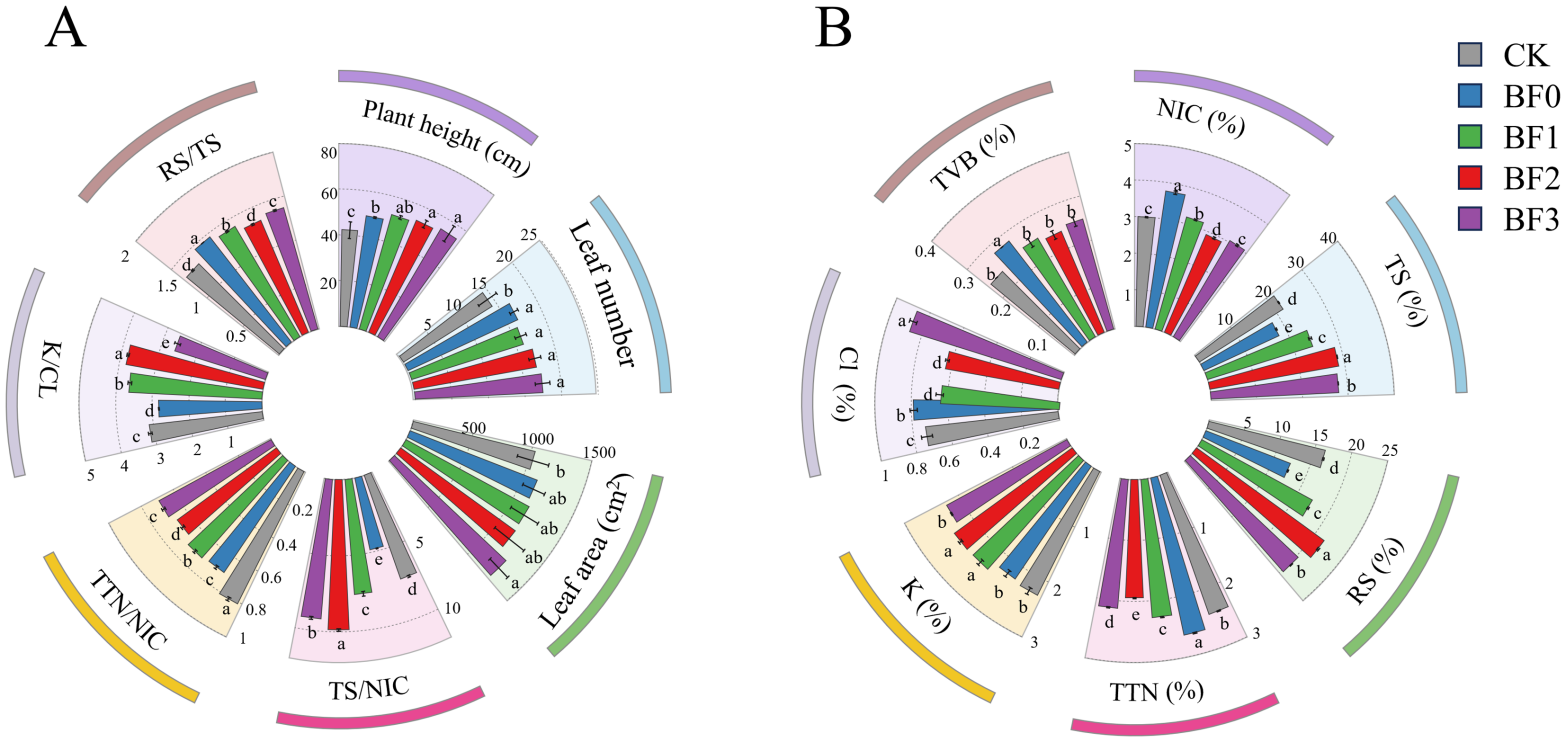
Agronomic traits of tobacco plants and chemical composition of tobacco leaves: **(A)** Agronomic traits of tobacco plants and coordination of chemical components, including plant height, leaf number, leaf area, sugar to nicotine ratio, nitrogen to nicotine ratio, potassium to chlorine ratio, and reducing-to-total sugar ratio. **(B)** Chemical components of tobacco leaves, including total sugar, reducing sugar, total nitrogen, potassium, chlorine, total volatile bases, and nicotine.

The changes in agronomic traits and chemical composition of tobacco plants (Figures 6A,B) indicated that the different treatments significantly affected plant growth, chemical components, and their coordination (*p* < 0.05). Regarding agronomic traits, compared with CK, the BF treatments generally promoted plant growth. Plant height under BF2 and BF3 treatments was significantly higher than that under CK (*p* < 0.05), with the highest value observed in BF3. The number of leaves per plant increased significantly across all BF treatments (*p* < 0.05). Leaf area also showed an increasing trend, with the largest value recorded under BF3, which was significantly higher than CK (*p* < 0.05). These results suggest that BF treatments substantially enhanced tobacco growth.

In terms of chemical composition, BF2 and BF3 treatments significantly increased the contents of TS and RS in tobacco leaves (*p* < 0.05), while NIC and TTN contents generally decreased. Meanwhile, BF1 and BF2 treatments significantly increased K content and reduced Cl content (*p* < 0.05). For coordination indices, BF2 treatment exhibited the highest TS/NIC and K/Cl, both significantly higher than CK (*p* < 0.05), along with the lowest TTN/NIC, indicating that this treatment effectively improved the chemical coordination of tobacco leaves.

### 3.6 Comprehensive evaluation of tobacco leaf quality

Correlation analysis of the indicators (Figure 1A) revealed extensive and complex interrelationships among the conventional chemical components of flue-cured tobacco, which simultaneously maintained a chemical balance among sugars, alkaloids, and nitrogenous compounds. TS and RS exhibited extremely significant negative correlations (*p* < 0.001) with NIC, and highly significant negative correlations (*p* < 0.01) with TVB. The TS/NIC showed a highly significant negative correlation (*p* < 0.01) with TTN, in addition to its expected correlations with sugar and alkaloid indicators, and a significant positive correlation with plant height. This indicates that the sugar content in leaves increased with plant biomass while maintaining a proportional balance with other nitrogenous and alkaloid indicators. The extremely significant negative correlations (*p* < 0.001) between TN and RS/TS, and the significant positive correlations (*p* < 0.05) of plant height with TS/NIC, RS, and TS, coupled with its significant negative correlation (*p* < 0.05) with TN, further illustrate this growth-balance relationship.

The weights of the 14 indicators in the comprehensive evaluation of tobacco leaves, determined using the entropy weight method, are shown in Figure 1B. The TS/NIC, RS, TS, and K/Cl were the top four indicators by weight, each exceeding 10%. This suggests substantial variation in these indicators among treatments, with the TS/NIC having the highest weight at 26.42%.

Following data normalization by the averaging method, an ideal matrix was constructed. The entropy weight method was used to assign weights to each indicator, followed by GRA. The bubble chart of GRA results (Figure 1C) shows that for the top four weighted indicators, the K/Cl in CK, TS in BF0, RS in BF1, and the TS/NIC in BF2 are represented by darker colors, indicating higher grey relational coefficients. After weighting, the TS/NIC ratio in the BF2 treatment had the highest weighted relational degree, meaning this indicator contributed most to the ideal sequence for that treatment.

The comprehensive relational coefficients (total scores) calculated by the GRA model for each treatment are shown in Figure 1D. The ranking of tobacco leaf quality across treatments from highest to lowest was BF2 > BF3 > BF1 > CK > BF0. No significant difference was found between the BF2 and BF3 treatment groups, and both treatments exhibited the highest overall leaf quality among all treatments.

After Z-score standardization of the quality indicators, NMDS analysis was performed (Figure 1E). The stress value was 0.073 (< 0.2), confirming the reliability of the ordination. The results show clear spatial separation among different treatment groups (CK, BF0, BF1, BF2, BF3) in the ordination plot. The control group CK was significantly separated from all BF treatment groups along the NMDS1 axis. The BF0, BF1, BF2, and BF3 groups also formed distinct clusters, with samples aggregating within groups and no overlap between groups, indicating that different treatments significantly altered the comprehensive quality of tobacco leaves.

Following Z-score standardization, a weighted matrix was constructed using the entropy weight method for TOPSIS analysis. The decision space plot (Figure 1F) visually displays the distances of each sample from the positive and negative ideal solutions in the TOPSIS method. The plot shows that CK samples are clustered near the relative closeness line of C_i_ = 0.5. The BF0 treatment is located on the side with C_i_ < 0.5, relatively far from the positive ideal solution and close to the negative ideal solution, indicating poorer performance. The BF1, BF3, and BF2 treatment groups are all distributed on the side with C_i_ > 0.5, being closer to the positive ideal solution while simultaneously distancing themselves from the negative ideal solution, with BF2 performing the best.

The relative closeness scores (Figure 1G) calculated by the TOPSIS model for each treatment confirmed that BF2 and BF3 performed best. Their ranking and significance trends were consistent with the results from the GRA model. Linear regression analysis (Figure 1H) between the two methods revealed a statistically significant positive linear relationship (*p* < 0.05) between the GRA comprehensive score and the TOPSIS comprehensive score, with a moderate explanatory power (R^2^ = 0.398).

### 3.7 Analysis of mechanisms underlying soil fertility and tobacco leaf quality enhancement

**Figure 7:**
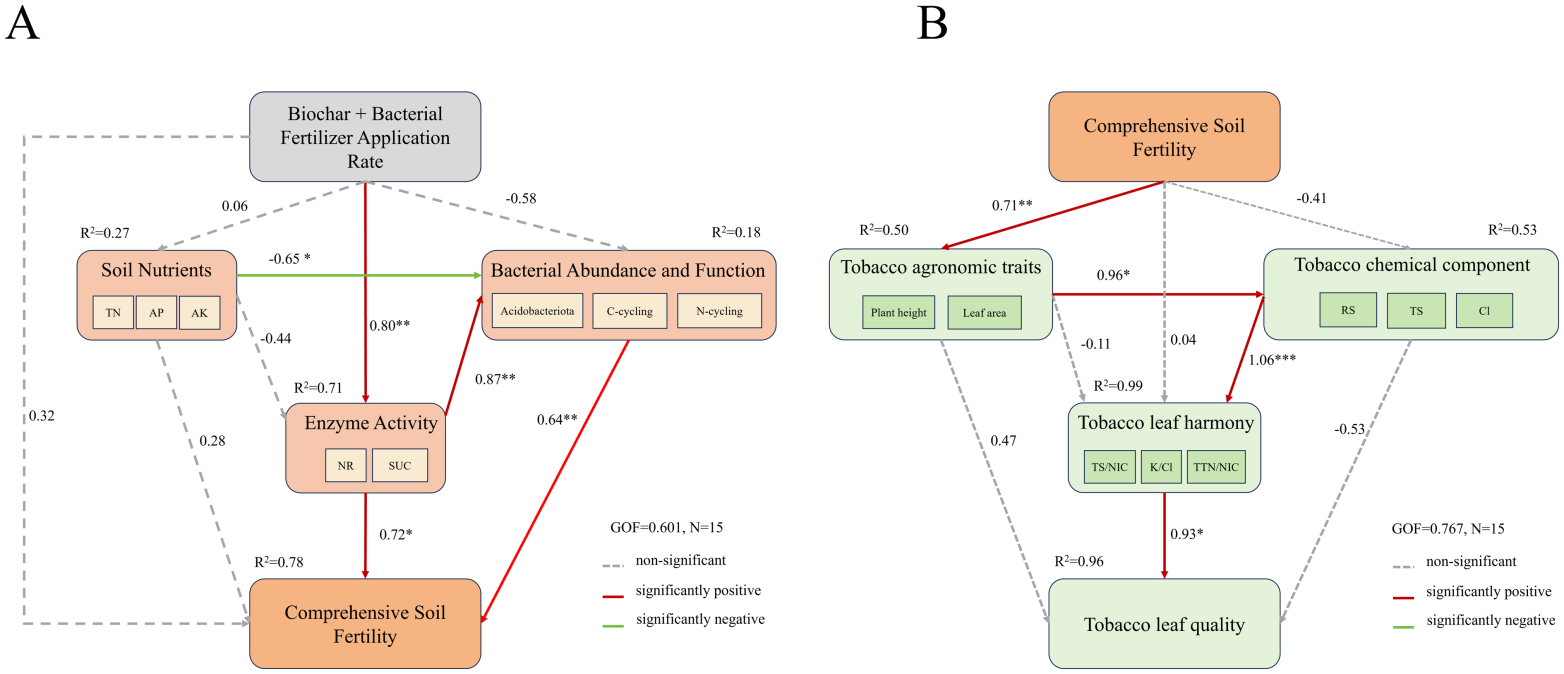
Partial least squares path modeling (PLS-PM): **(A)** Pathways influencing soil fertility, **(B)** pathways enhancing tobacco leaf.

Based on partial least squares path modeling (PLS-PM), the relationships among soil nutrients (TN, AP, AK), enzyme activities (NR, SUC), microbial abundance and function (Acidobacteriota, C-cycling, N-cycling), and comprehensive soil fertility under biochar and microbial fertilizer co-application treatments were analyzed (Figure 7A). The model exhibited a good overall fit (GOF = 0.601), explaining 78% of the variance in comprehensive soil fertility. The results indicated significant paths between the co-application of biochar and microbial fertilizer and soil enzyme activity, with a highly significant direct effect (path coefficient = 0.80, *p* < 0.01). Changes in soil enzyme activity, in turn, directly and significantly affected comprehensive soil fertility (path coefficient = 0.72, *p* < 0.05). Furthermore, alterations in enzyme activity exerted a highly significant influence on soil microbial abundance and function (path coefficient = 0.87, *p* < 0.01), which subsequently had a significant indirect effect on comprehensive soil fertility (path coefficient = 0.64, *p* < 0.01).

Another PLS-PM was constructed to analyze the relationships among changes in comprehensive soil fertility, tobacco plant agronomic traits (Plant height, Leaf area), flue-cured tobacco chemical properties (RS, TS, Cl), leaf compositional harmony (TS/NIC, K/Cl, TTN/NIC), and final leaf quality (Figure 7B). This model also showed a good overall fit (GOF = 0.767), explaining 96% of the variance in tobacco leaf quality. The results demonstrated that changes in comprehensive soil fertility had a highly significant direct effect on tobacco plant agronomic traits (path coefficient = 0.71, *p* < 0.01). Variation in agronomic traits significantly influenced leaf chemical properties (path coefficient = 0.96, *p* < 0.05). Changes in chemical properties directly affected leaf compositional harmony (path coefficient = 1.06, *p* < 0.001), and finally, alterations in compositional harmony had a significant direct impact on the final tobacco leaf quality (path coefficient = 0.93, *p* < 0.05).

## 4 Discussion

### 4.1 Synergistic regulation of acidified soil fertility through biochar-microorganism interaction

This study demonstrates that the synergistic application of biochar and *Bacillus subtilis* fundamentally reshapes the regulatory pathway of soil fertility in acidified systems, shifting it from predominantly physicochemical regulation to a microbially mediated process. Rather than acting independently, biochar and microorganisms function as a coupled system in which biochar provides ecological niches and stabilization effects, while microbial activity drives enzymatic transformations and nutrient cycling. Notably, our results reveal that soil enzyme activity serves as a central regulatory hub linking microbial functional shifts with improvements in soil fertility. This mechanism highlights that the effectiveness of soil amendment strategies depends not only on direct changes in soil properties, but more critically on their capacity to activate microbial-mediated biogeochemical processes.

With the advancement of soil microbial ecology theory, the understanding of the rhizosphere microbiome as a core driver at the plant–soil interface has deepened (Trivedi et al., 2020). Strategies for managing agricultural soil acidification have shifted from relying on single physicochemical indicators to systematic regulation integrated with microbial processes (Xu et al., 2023). Rhizosphere microorganisms maintain system stability and functionality by regulating key processes such as nutrient cycling, energy flow, and signal transduction (Pandey and Saharan, 2025). Applying microbial regulation to improve soil quality and crop yield has become a significant research direction for sustainable agriculture, with its core focus on optimizing microbial community structure to enhance ecosystem service functions (Zhao et al., 2022).

Traditional amendments for acidic soils often emphasize using lime to directly neutralize acidity or adding organic matter to improve physical structure, but these measures have limited effect on activating the soil biological network (Lu et al., 2025). Even biochar, possessing certain biocompatibility, often acts as a slow-release physicochemical amendment when applied alone. While it can enhance soil water and nutrient retention, its direct regulatory effect on indigenous microbial community structure and function is typically limited, and its efficacy is strongly modulated by soil type, crop root activity, and environmental factors (Iturbe-Espinoza et al., 2025; Fakhar et al., 2025). In this study, the sole application of biochar increased soil pH and alleviated acidification, but offered limited improvement in overall soil fertility or crop quality, indicating that purely physicochemical amendments may have inherent limitations (Wang et al., 2020). In contrast, introducing *B. subtilis* allows biochar to serve as a carrier and protective matrix. The metabolic activities of the microorganism can directly participate in nutrient mobilization and may indirectly and synergistically regulate the soil microenvironment by accelerating biochar surface transformation and release of functional groups, thereby improving soil properties across broader dimensions. Thus, the biochar-microorganism combination integrates physical amendment with bioactive regulation, acting on both abiotic and biotic soil attributes for more stable and lasting improvement.

Soil quality is the material foundation of agricultural production, and the microbial community influences soil fertility and health through its structural characteristics and functional activity (Lehmann et al., 2020). Biochar combined with *B. subtilis* was applied to ameliorate acidic soil, and it was found that exogenous bacteria activated microbial metabolism and indirectly regulated pH (Diaz-Chuquizuta et al., 2025). The significant differences in pH effects between sole biochar and the combination confirmed the key role of microorganisms. Nutrient cycling is one of the most fundamental ecological processes in ecosystems, where microbe-driven biogeochemical cycles directly affect nutrient availability and utilization efficiency (Jansson and Hofmockel, 2020). This study demonstrated that biochar-microorganism co-application exhibited significant combined effects in enhancing soil nutrient availability. Different microbial fertilizer treatments had varied impacts: BF1 significantly increased TN, AN, and AP, while AP decreased under BF2 and BF3; BF3 also showed the most pronounced increase in AK. The decrease in AP observed under BF2 and BF3 may be related to enhanced plant uptake and microbial immobilization under improved growth and microbial activity, rather than a decline in soil fertility per se. When considered together with the elevated AK in BF3 and the overall beneficial effects of these treatments, this pattern suggests that co-application maintained or even improved nutrient supply capacity (Sun et al., 2020).

The increase in CEC further confirmed the mechanism by which biochar enhances soil nutrient retention through its developed pore structure and abundant functional groups (Munera-Echeverri et al., 2018). Regarding enzyme activities, the highly significant increase in NR and SUC activities, most prominent in BF2, indicated that biochar-microorganism interaction strongly stimulated soil carbon and nitrogen transformation functions (Wang et al., 2023). This aligns with the growth-promoting and metabolic characteristics of Bacillus strains, which participate directly in nutrient mobilization by secreting extracellular enzymes, while biochar can adsorb enzyme proteins, prolonging their functional persistence (Girelli et al., 2020; Nie et al., 2025). The results further clarify the non-linear impact of microbial fertilizer dosage on enzyme activity response: an appropriate dosage (BF2) maximally activated microbial metabolism, while an excessive amount (BF3) might lead to nutrient competition or accumulation of metabolic by-products, potentially inhibiting certain enzyme activities.

### 4.2 Response of bacterial community structure and function to biochar-microorganism co-application in acidified soil: the mediating role of enzyme activity

In acidic soils, nitrification is often inhibited, and ammonia-oxidizing bacteria activity is reduced, leading to decreased nitrogen transformation efficiency and insufficient plant-available nitrogen supply (Li et al., 2018). In this study, the co-application of biochar and microorganisms enhanced the soil microbial predicted nitrogen cycling potential and function, likely by promoting nitrification and ammonification efficiency, thereby increasing nitrogen bioavailability (Liu et al., 2019). Additionally, the improved microbial community contributed to driving the cycling and mobilization of carbon, phosphorus, and other elements, gradually ameliorating soil nutrient balance. Thus, the biochar-microorganism interaction not only modulates soil physicochemical properties in the short term but, more importantly, enhances the self-regulation and nutrient supply capacity of the soil ecosystem from the perspective of microbial community structure and function, providing a functional foundation for ecological restoration and sustainable fertility enhancement of acidic soils.

Early research indicated that biochar application can directly shape soil microbial function through multiple pathways. Its alkalizing effect can modify the activity of ammonia-oxidizing microorganisms and thereby influence nitrification processes (Zheng et al., 2022); its abundant pore structure can create anaerobic microsites that specifically enrich denitrifying microorganisms (Zhu et al., 2022); simultaneously, its input fundamentally alters the soil C: N ratio and substrate availability, driving microbial community structure toward a new equilibrium (Li et al., 2026). In addition, biochar can function as a microhabitat that facilitates microbial colonization and persistence. Its highly porous structure and large surface area provide protective niches that buffer microorganisms from environmental stress while retaining nutrients and organic substrates. These microsites may enhance microbial interactions and promote the establishment of functional taxa involved in carbon and nitrogen cycling, thereby strengthening microbial-mediated ecosystem processes.

Our study further reveals that co-application significantly optimized soil bacterial community structure and likely reinforced carbon and nitrogen transformation processes by enhancing key enzyme activities, directionally promoting the enrichment of functional microorganisms. The BF2 treatment performed best across all alpha diversity indices, suggesting that a moderate dosage of microbial fertilizer is most conducive to maintaining and enhancing microbial diversity. Concurrently, functional genera like *Sphingomonas* and *Arthrobacter*, known for degrading organic matter and promoting nutrient cycling, were significantly enriched in this treatment (Qu et al., 2023). The prosperity of these functional groups is key to driving soil nutrient transformation efficiency (Zheng et al., 2025). NMDS and ANOSIM analyses confirmed that co-application altered the soil microbiome. By providing habitat niches and resource heterogeneity, biochar likely increased the scale and complexity of microbial co-occurrence networks; when combined with functional microorganisms, this regulation can enhance interactions within functionally related microbial groups (Yan et al., 2021; Ma et al., 2025; Tao et al., 2025).

Collectively, PLS-PM analysis establishes a hierarchical mechanism linking biochar–microbe co-application to crop quality, wherein enzyme activity serves as a central hub mediating the effects of amendments on microbial function and soil fertility. These enzyme-driven processes shape microbial functional composition—particularly nitrogen cycling—which indirectly enhances soil fertility. A second model further reveals a cascading pathway from soil fertility to plant growth, leaf chemical composition, and ultimately crop quality, with the balance of chemical components governing quality formation. This stepwise soil–plant coupling pathway supports that biochar–microbe interactions drive quality improvement through enzyme-mediated microbial regulation.

### 4.3 Formation response of tobacco leaf quality and its comprehensive evaluation method

Tobacco leaf quality is influenced by multiple factors, including genetic background, ecological environment, and cultivation practices. Its formation is essentially a comprehensive manifestation of the plant’s metabolism and accumulation under complex interactions (Xiao et al., 2025). The composition and ratio of chemical components in tobacco leaves serve as the core basis for defining their intrinsic quality, with the content and harmony of key indicators holding clear agronomic significance. The K/Cl directly relates to combustion properties, the TS/NIC reflects the balance between aroma quality and irritation, and the TTN/NIC indicates the smoothness of smoke (Yang et al., 2026). Therefore, achieving coordinated optimization of key chemical components is a common goal in high-quality tobacco production. Nonetheless, the comprehensive evaluation of tobacco leaf quality faces significant challenges. Compared to most field crops, while tobacco quality research has a longer history and a relatively systematic indicator framework, its evaluation process is more complex. This stems both from the widespread correlations and non-linear interactions among chemical indicators, and from the fact that these key indicators have dynamic optimal ranges depending on the producing region’s ecology, cultivar characteristics, and industrial requirements. This makes it difficult to establish a unified and objective evaluation standard, and the related theoretical system and evaluation methods are still evolving (Zhai et al., 2022; Yang et al., 2025).

In this study, the improvement in leaf quality originated from the optimization of the soil microenvironment. Sole biochar application increased nitrogen and nicotine alkaloids content but led to an imbalance in the TS/NIC, while excessive microbial fertilizer caused Cl accumulation and a decline in the K/Cl. This indicates the existence of an optimal co-application ratio between biochar and microbial fertilizer. Biochar fixes soil chloride ions through adsorption, reducing their translocation into the plant (Yu et al., 2019), whereas *B. subtilis* promotes potassium uptake by mobilizing soil potassium and playing key roles in carbon and nitrogen metabolism (Peng et al., 2026). The BF2 treatment, by optimizing the rhizosphere microenvironment, performed best in coordinating leaf chemical composition, achieving the characteristics of high sugar, high potassium, and low chlorine, with its TS/NIC and K/Cl falling within harmonious ranges (Wang et al., 2022). Research indicates extensive significant correlations among multiple chemical indicators. Although many indicators have recognized optimal ranges, these ranges are subjectively adjusted based on region, cultivar., and target product style (Jing et al., 2024). This makes traditional PCA less adequate for handling the non-linear relationships between indicators and the problem of nominal-the-best value orientation. To address this, this study employed the entropy weight method to determine indicator weights and combined it with GRA for quality evaluation. GRA is suitable for comprehensive evaluation problems with small sample sizes, limited information, and unclear relationships between indicators. However, the subjectively set optimal reference sequence in GRA can still affect the objectivity and universality of the results. Therefore, TOPSIS was used for cross-validation. This method evaluates the superiority of options by calculating their geometric distances to the positive and negative ideal solutions generated from the actual data itself. Its core ranking mechanism does not rely on a pre-defined fixed reference sequence, thus providing an alternative comprehensive ranking perspective based on relative distances, which can effectively compare and complement the GRA results. Notably, the ranking trends of treatments by GRA and TOPSIS were highly consistent, providing excellent mutual validation of result reliability. The main reason for this consistency likely lies in the fact that, despite different algorithmic principles, both methods are based on the same data matrix and effectively capture the overall proximity of each treatment’s leaf chemical composition to an ideal state, thereby reaching a consensus on the core trend reflecting differences in comprehensive quality.

## 5 Conclusion

Compared with conventional fertilization, the application of rice husk biochar (1,500 kg·ha^−1^), combined with *B. subtilis*, significantly improved soil fertility in acidified red soils by enhancing soil physicochemical properties, stimulating enzyme activities, and reshaping soil bacterial communities. Although BF1 and BF2 showed similarly high soil fertility, and BF2 and BF3 exhibited comparable tobacco leaf quality, BF2 (30 kg·ha^−1^) achieved the best overall balance between soil fertility improvement and leaf quality optimization, likely due to its ability to enhance microbial diversity and functional potential related to carbon and nitrogen cycling, and is therefore recommended as the optimal management strategy.

Improved soil fertility subsequently enhanced tobacco growth and optimized leaf chemical composition, thereby improving tobacco leaf quality. Overall, these findings highlight that biochar-microbe interactions play a key role in regulating soil microbial processes in acidified soils. The combined application of biochar and *B. subtilis*, particularly at moderate microbial dosage, represents a promising strategy for improving soil health and crop quality in acidified agricultural systems.

## Data Availability

The datasets presented in this study can be found in online repositories. The names of the repository/repositories and accession number(s) can be found in the article/Supplementary material.

## Footnotes

1
https://www.majorbio.com
