# Supplementary material for: Synergistic effects of biochar and Bacillus subtilis on soil fertility and tobacco leaf quality in acidified soil: microbial mechanisms and multi-model evaluation
Source: Front Microbiol. 2026 Jun 9;17:1842613. doi: 10.3389/fmicb.2026.1842613 (PMC13286961; doi:10.3389/fmicb.2026.1842613)
Supplement: Supplementary file 1 [file Data_Sheet_1.DOCX]

Supplementary Material

This supplementary material includes three tables and one figure:

- Table S1 Basic chemical properties of the tested soil.
- Table S2 Basic chemical properties of the rice husk biochar used in this study.
- Table S3 Chemical Standards for Premium Flue-cured Tobacco (Middle Leaves).
- Figure S1 Heatmap of factor loadings of soil indicators on PC1–PC4.

Further inquiries and requests for materials should be directed to the corresponding author: Bo Li (libo@ynau.edu.cn) or Jincheng Ao (ecojin2023@163.com).

Tables S1 Basic chemical properties of the tested soil.

| Parameter (Unit) | Value |
| --- | --- |
| pH | 5.45 |
| Organic matter (g·kg^-1^) | 27.58 |
| Alkali-hydrolyzable N (mg·kg^-1^) | 52.50 |
| Available P (mg·kg^-1^) | 57.70 |
| Available K (mg·kg^-1^) | 235.00 |
| Exchangeable Ca (mg·kg^-1^) | 586.31 |
| Exchangeable Mg (mg·kg^-1^) | 61.82 |
| Water-soluble Cl⁻ (mg·kg^-1^) | 7.80 |
| Available B (mg·kg^-1^) | 1.22 |
| Total N (g·kg^-1^) | 0.35 |
| Total P (g·kg^-1^) | 2.70 |

Tables S2 Basic chemical properties of the rice husk biochar used in this study.

| Parameter (Unit) | Value |
| --- | --- |
| pH | 9.67 |
| Organic matter (%) | 31.28 |
| Total N (g·kg^-1^) | 4.83 |
| Total P (g·kg^-1^) | 1.19 |
| Total K (g·kg^-1^) | 15.63 |
| Alkali-hydrolyzable N (mg·kg^-1^) | 6.42 |
| Available P (mg·kg^-1^) | 357.81 |
| Available K (mg·kg^-1^) | 12.28 |

Tables S3 Chemical Standards for Premium Flue-cured Tobacco (Middle Leaves)

| Chemical Component | Suitable Range (%) |
| --- | --- |
| TS | 24~35 |
| RS | 19.0~20.0 |
| TTN | 2.0~2.3 |
| NIC | 1.5~3.5 |
| TVB | 0.30~0.35 |
| TS/NIC | 8.5~9.5 |
| K/Cl | 0.8~1.2 |

**
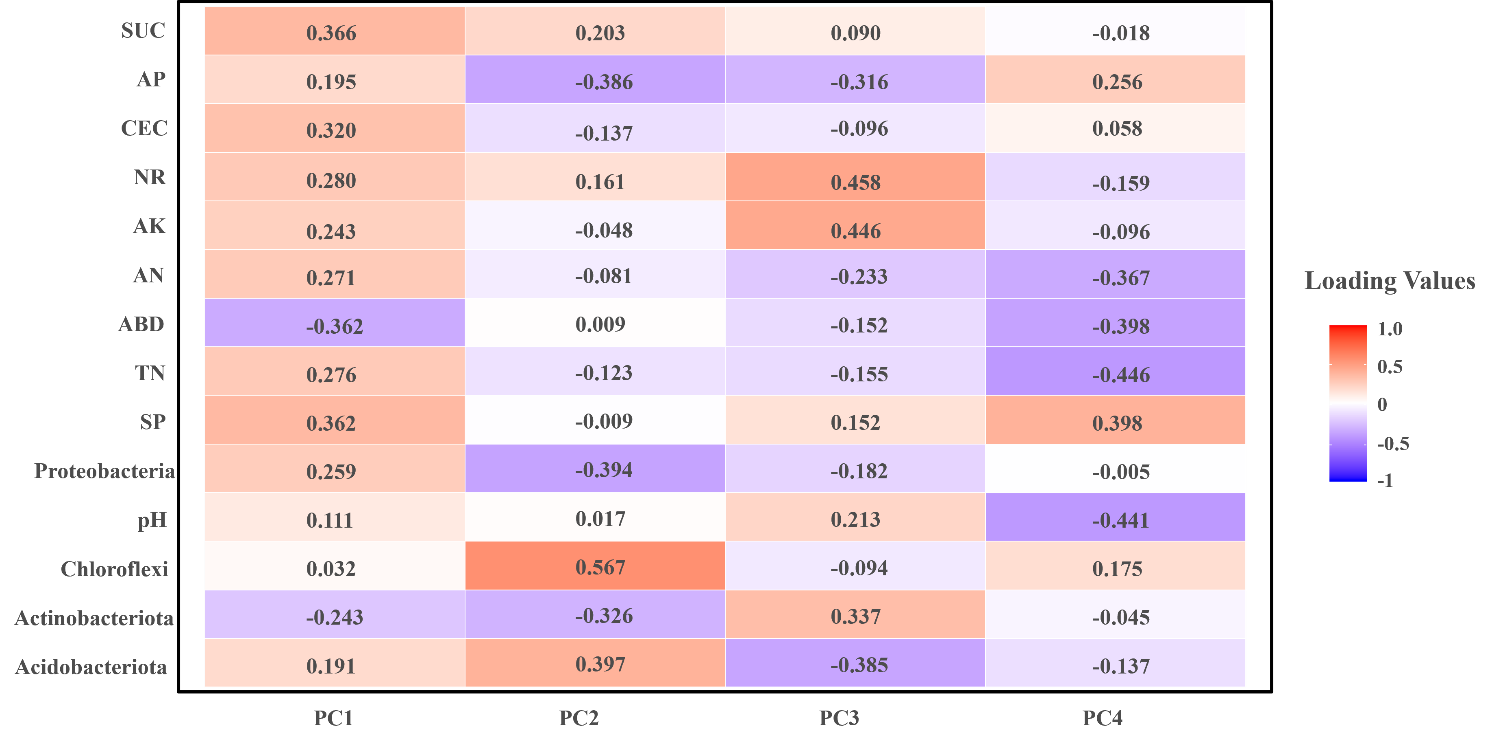
**

FIGURE S1. Heatmap of factor loadings of soil indicators on PC1–PC4.
